# Napoleon Bonaparte—A Possible Case of Trench Fever

**DOI:** 10.3201/eid3105.240970

**Published:** 2025-05

**Authors:** Éric Faure

**Affiliations:** I2M–CNRS UMR 7373, Aix-Marseille University, Marseilles, France

**Keywords:** Napoleon Bonaparte, trench fever, Bartonella quintana, Pediculus humanus corporus, vector-borne infections, bacteria, France

## Abstract

In 1789, Napoleon Bonaparte reported having a recurrent febrile illness that initially subsided for 4 days and then had multiple relapses of similar duration. A speculative diagnosis of trench fever would be supported by poor hygiene conditions, prolonged exposure to cold, and the presence of lice in Napoleon’s barracks environment.

Napoleon Bonaparte’s health has been the subject of numerous retrospective studies. He has been thought to have had ≈40 individual diseases (*1*), and an additional disease should be added to that list. In January 1789, Bonaparte, who served at the Artillery School of Auxonne, France, wrote to his mother: “I have had attacks of persistent fever from time to time. The fever subsides for some four days and then relapses, lasting for about the same time again” (*2*).

Auxonne was notoriously malarious, and Bonaparte, who had already had intermittent fevers, attributed his illness to miasmas, noxious forms of “bad air” (*2*,*3*). However, the periodicity of his fevers would rather suggest a more likely diagnosis of trench fever, a disease caused by *Bartonella quintana*, a bacteria transmitted by infected human body lice. The fever pattern of trench fever is frequently characterized by episodes of fever lasting 4–5 days, with apyretic intervals of 4–5 days between each episode (*4*). The major predisposing risk factors for trench fever are poor hygiene, louse infestation, immune system compromise, and prolonged exposure to cold (*5*), all factors likely present at the time of Napoleon’s correspondence. The winter of 1788–89 was extreme. Bonaparte had been ill during the summer of 1788 (*3*,*6*). In March 1789, Bonaparte wrote that he rarely changed his clothes, which was a habit he had practiced in the past (*3*). The presence of lice at the School of Auxonne was likely (*3*).

Trench fever was not recognized as a distinct clinical entity until 1915 (*7*). Nonetheless, analysis of DNA extracted from gravesites has provided evidence of human infection with *B. quintana* since at least 4,000 years ago (*8*). Researchers have identified *B. quintana* frequently in both barrack and field environments and have detected its DNA in mass graves of soldiers from the Napoleonic era in Vilnius, Lithuania, and in Kassel, Germany (*8*).

Retrospective diagnosis is always a precarious endeavor, particularly without DNA evidence. However, I believe that trench fever should be recognized as among the many ailments that befell Napoleon Bonaparte.
